# Assessment of the cancer risk factors of solitary pulmonary nodules

**DOI:** 10.18632/oncotarget.16426

**Published:** 2017-03-21

**Authors:** Li Yang, Qiao Zhang, Li Bai, Ting-Yuan Li, Chuang He, Qian-Li Ma, Liang-Shan Li, Xue-Quan Huang, Gui-Sheng Qian

**Affiliations:** ^1^ Interventional Radiology Department, the First Hospital of the Third Military Medical University, Chongqing 400038, China; ^2^ Institute of Respiratory Diseases, the Second Hospital of the Third Military Medical University, Chongqing 400038, China

**Keywords:** solitary, pulmonary nodule, malignancy, risk factor, model

## Abstract

There are no large samples or exact prediction models for assessing the cancer risk factors of solitary pulmonary nodules (SPNs) in the Chinese population. We retrospectively analyzed the clinical and imaging data of patients with SPNs who underwent computer tomography guided needle biopsy in our hospital from Jan 1st of 2011 to March 30th of 2016. These patients were divided into a development data set and a validation data set. These groups included 1078 and 344 patients, respectively. A prediction model was developed from the development data set and was validated with the validation data set using logistic regression. The predictors of cancer in our model included female gender, age, pack-years of smoking, a previous history of malignancy, nodule size, lobulated and spiculated edges, lobulation alone and spiculation alone. The Area Under the Curves, sensitivity and specificity of our model in the development and validation data sets were significantly higher than those of the Mayo model and VA model (*p* < 0.001). We established the largest sampling risk prediction model of SPNs in a Chinese cohort. This model is particularly applicable to SPNs > 8 mm in size. SPNs in female patients, as well as SPNs featuring a combination of lobulated and spiculated edges or lobulated edges alone, should be evaluated carefully due to the probability that they are malignant.

## INTRODUCTION

With increases in the clinical utilization of computed tomography (CT) in recent years, the pulmonary nodule detection rate has increased tremendously [1–3]. In several large screening studies of lung cancer, the pulmonary nodule detection rate has increased from 8 to 51%. Malignant nodules account for 1.1 to 12% of these nodules [2]. A solitary pulmonary nodule (SPN) is a single, round, well-circumscribed radiological opacity ≤ 3 cm in diameter [4]. Most SPNs are detected incidentally by chest radiography and CT during investigations of other diseases [5]. The great diagnostic challenge facing clinicians in the evaluation of a patient with an SPN is the definitive establishment of whether the nodule is benign or malignant. Fleishner and the American College of Chest Physicians (ACCP) guidelines recommend that nodules 8 mm or smaller undergo mandatory follow-up CT evaluations based on their diameters and associated risk factors. The guidelines also recommend that individuals with nodules larger than 8 mm undergo a diagnostic work-up consisting of more invasive diagnostic procedures [4, 6–8]. Devising effective strategies for managing patients with SPNs depends critically on the pre-test probability of malignancy [9]. However, there are no large samples or exact prediction models for assessing the cancer risk factors of SPNs, especially in the Chinese population. Moreover, it is important to highlight that existing studies have not used more advanced imaging modalities to characterize pulmonary nodules in their analyses [5].

The aim of our study was to screen the risk factors for malignant SPNs, establish a risk prediction model from a sample of consecutive patients (*n* = 1078) and test the model on data from a separate group of patients (*n* = 344) with SPNs who underwent CT-guided needle biopsies in our hospital. We also evaluated the accuracy and calibration of the Mayo Clinic and VA models, which were developed using North American or European populations to estimate the probability of malignancy, to determine whether any differences existed between our model and the Mayo and VA models with respect to cancer risk factors.

## RESULTS

A total of 1422 consecutive patients with SPNs were enrolled in this study. The development data set included 1078 patients who were evaluated from Jan 1^st^ of 2011 to April 30^th^ of 2015, and a separate validation data set included 344 patients who were evaluated from May 1^st^ of 2015 to March 30^th^ of 2016. All patients enrolled in the study had biopsy pathology results. Twenty patients and four patients in the development and validation data sets, respectively, underwent repeat biopsies, and 186 patients and 24 patients in the development and validation data sets, respectively, had surgical pathology results.

### Clinical data

Demographic data: In the development data set (647 men and 431 women aged 17–87 years, mean 55.41 ± 11.94 years), 414 patients had a history of smoking, and 54 patients had a history of cancer (Table 1). Additional patient clinical features are shown in Supplementary Table 1. In the validation data set (196 men and 148 women aged 13–85 years, mean 55.34 ± 11.24 years), 112 patients had a history of smoking, and 16 patients had a history of cancer. There were no significant differences between the two groups with respect to the above data (*p* > 0.05) (Table 1).

**Table 1:** Clinical characteristics of the SPNs in development and validation data sets

Characteristics	Development data set, *n* (%)					Validation data set, *n* (%)				p
	Total	Malignant	Benign	Non-diagnostic		Total	Malignant	Benign	Non-diagnostic	
Participants	1078	721	182	175		344	236	46	62	
Males	647	439	98	110		196	132	23	41	0.317
Females	431	282	84	65		148	104	23	21	
Age (years)	55.41 ± 11.94	58.22 ± 10.83	49.01 ± 11.88	50.53 ± 12.27		55.34 ± 11.24	57.48 ± 10.22	47.17 ± 12.02	53.27 ± 11.35	0.105
Smoking status										
Censored data	249	163	39	47		78	42	15	21	0.871
Non-smoker	415	269	84	62		154	111	22	21	0.000
Former or current smoker	414	289	59	66		112	83	9	20	0.051
Pack-year	38.95 ± 39.71	41.03 ± 36.58	36.09 ± 63.51	32.39 ± 20.50		35.81 ± 20.63	34.95 ± 20.55	36.78 ± 26.54	38.95 ± 18.76	0.081
< 30 (n,%)	139	80	30	29		33	26	3	4	0.411
≥ 30 (n,%)	275	209	29	37		79	57	6	16	
Previous medical history										
Censored data	217	139	34	44		72	41	12	19	0.748
No disease	479	317	89	73		191	144	21	26	0.000
Extra-thoracic disease	176	121	32	23		46	27	9	10	0.189
Lung disease excluding malignancy	152	99	20	33		19	14	4	1	0.000
Malignancy	54	45	7	2		16	10	0	6	0.789

SPN CT characteristics: In the development data set, the average size of the SPNs was 18.43 ± 5.03 mm (ranging from 4.625 mm to 29.965 mm), and 98.42% of SPNs were > 8 mm. In the validation data set, the average size of the SPNs was 18.16 ± 5.05 mm (ranging from 5.87 mm to 29.515 mm), and 97.67% of SPNs were > 8 mm. There were no significant differences between the two groups with respect to SPN size (*p* > 0.05) (Table 2).

**Table 2:** CT characteristics of the SPNs in the development and validation data sets

CT Characteristics	Development data set, *n* (%)				Validation data set, *n* (%)				p
	Total	Malignant	Benign	Non-diagnostic	Total	Malignant	Benign	Non-diagnostic	
Size (mm)	18.43 ± 5.03	19.41 ± 4.89	16.47 ± 4.54	16.44 ± 4.91	18.16 ± 5.05	19.28 ± 4.66	16.71 ± 4.84	14.97 ± 5.09	0.388
4-8 mm	17	5	4	8	8	2	0	6	0.358
8-10 mm	55	24	15	16	16	7	4	5	0.738
10-20 mm	632	388	129	115	190	118	31	41	0.267
20-30 mm	374	304	34	36	130	109	11	10	0.296
Edge									
Spiculated Protuberances	244	153	47	44	88	62	11	15	0.088
Lobulation	363	298	33	32	70	60	3	7	0.000
Spiculation	234	161	34	39	73	46	11	16	0.849
Lobulation and spiculation	83	77	4	2	35	34	1	0	0.147
Irregular edge	78	21	22	35	46	26	8	12	0.000
Smooth edge	76	11	42	23	32	8	15	9	0.170
Density									
Solid	728	515	121	92	190	135	25	30	0.000
Purely ground-glass	3	3	0	0	6	4	0	2	0.003
Partly solid	170	117	18	35	105	18	8	19	0.000
Thin cavitation	5	3	1	1	0	0	0	0	0.206
Thickened cavitation	116	75	20	21	26	17	5	4	0.085
Necrosis	14	6	2	6	5	1	4	0	0.828
Calcification	42	2	20	20	12	1	4	7	0.730
Location									
Upper lobe	596	409	90	97	202	150	22	30	0.264
Middle lobe	114	85	18	11	32	21	5	6	
Lower lobe	368	227	74	67	110	65	19	26	

**Figure 1:**
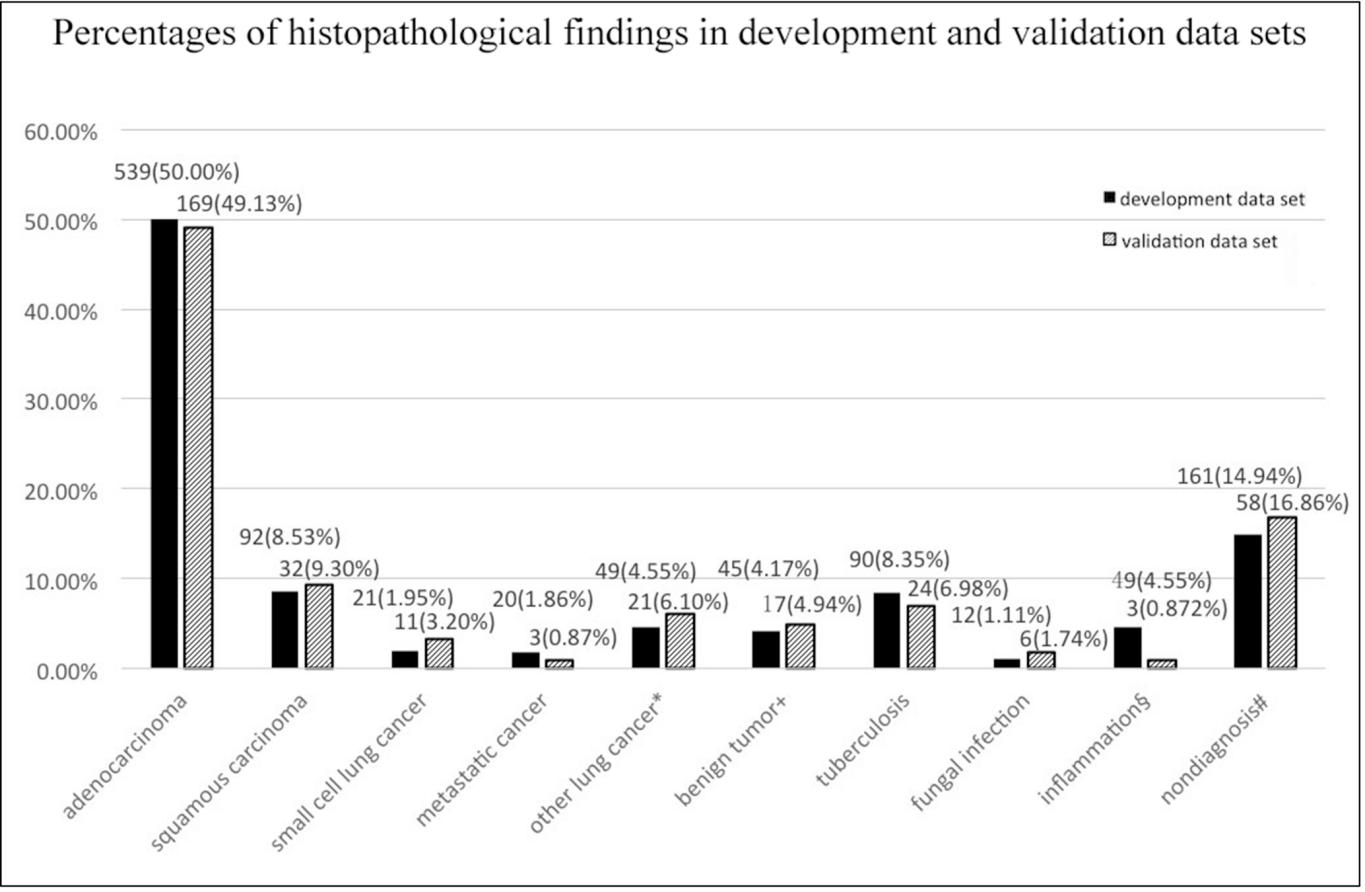
Histopathology of the development and validation data *The development data set comprised 24 non-small cell lung cancers, 6 neuroendocrine carcinomas, 4 lymphoma-like epithelial carcinomas, 4 complex carcinomas, 8 carcinoids, 2 sarcoma carcinomas, and 1 lymphoma. The validation data set comprised 19 NSCLCs, 1 neuroendocrine carcinoma, and 1 sarcoma carcinoma. ^†^The development data set comprised 16 sclerosing angiomas, 11 hamartomas, 8 cartilaginous tumors, 2 vascular smooth muscle tumors, 6 inflammatory pseudotumors, and 2 spindle cell tumors. The validation data set comprised 4 sclerosing angiomas, 3 hamartomas, 3 inflammatory pseudotumors, 3 spindle cell tumors, 2 bronchoceles and 2 chondrophymas. ^§^In the development data set, 29 nodules became markedly smaller within a short time, and 20 cases were stable after more than two years. The validation data set comprised 2 suppurative inflammation lesions and 1 eosinophilic inflammatory lesion. ^#^ Conditions that did not have a final diagnosis, including chronic inflammation, fibroplasia and gland hyperplasia.

Histopathology: In the development data set, 721 cases were malignant lesions (66.883%), 196 cases were benign lesions (18.182%), and 161 cases were non-diagnostic results (14.935%). Among the 721 malignant cases, 5 were diagnosed by repeat lung biopsy, and 3 were diagnosed by surgical resection. In the validation data set, 236 cases were malignant lesions (68.605%), 50 cases were benign lesions (14.535%), and 58 cases were non-diagnostic results (16.860%) (Figure 1). There were no significant histopathological differences between the two groups (*p* > 0.05).

### Logistic regression analysis of the risk factors for malignant SPNs

Female gender (OR 3.893, 95% CI 1.372–2.319, *p* < 0.001), age (OR 1.784, 95% CI 1.858–8.155, *p* < 0.001), pack-years of smoking (OR 1.756, 95% CI 1.139–2.707, *p* < 0.05), a previous history of malignancy (OR 3.382, 95% CI 1.512–6.283, *p* < 0.05), nodule size (OR 2.319, 95% CI 1.494–3.599, *p* < 0.001), lobulated and spiculated edges (OR 13.433, 95% CI 2.512–71.833, *p* < 0.001), lobulation alone (OR 2.203, 95% CI 1.100–4.416, *p* < 0.05) and spiculation alone (OR 1.556, 95% CI 0.766–3.162, *p* < 0.05) were risk factors for malignant SPNs, whereas irregular edges (OR 0.276, 95% CI 0.101–0.753, *p* < 0.05) and calcification (OR 0.106, 95% CI 0.028–0.410, *p* < 0.05) were protective factors for malignant SPNs. Symptom history, family history and upper lobe location were not associated with a risk of malignant SPNs.

### Equations for models estimating the pre-test probability of malignant SPNs

Based on the published literature, we adopted the following predictive mathematical models to estimate the malignant probability, with x varying in accordance with different formulas, such as *p* = e^x^ / (1+e^x^)where e is the natural logarithm, and qualitative factors, including gender (male = 1, female = 2), previous medical history (1 = yes, 0 = no), edge (1 = yes, 0 = no), and calcification (1 = yes, 0 = no).

We used the following equation in our model: X = −6.173+1.207*Gender+0.580*Age (years) + 0.520*Pack-year-0.226*Previous extrathoracic disease-0.685*Previous chronic lung disease except cancer+2.739*Malignancy history+0.933*Diameter (mm) + 0.702*Lobulation + 0.466*Spiculation+ 21.060*Lobulation and Spiculation-1.428*Irregular edges-2.062*Calcification.

### Receiver operating characteristic (ROC) curve analysis of the Area Under the Curves (AUC values) of the prediction models

**Figure 2:**
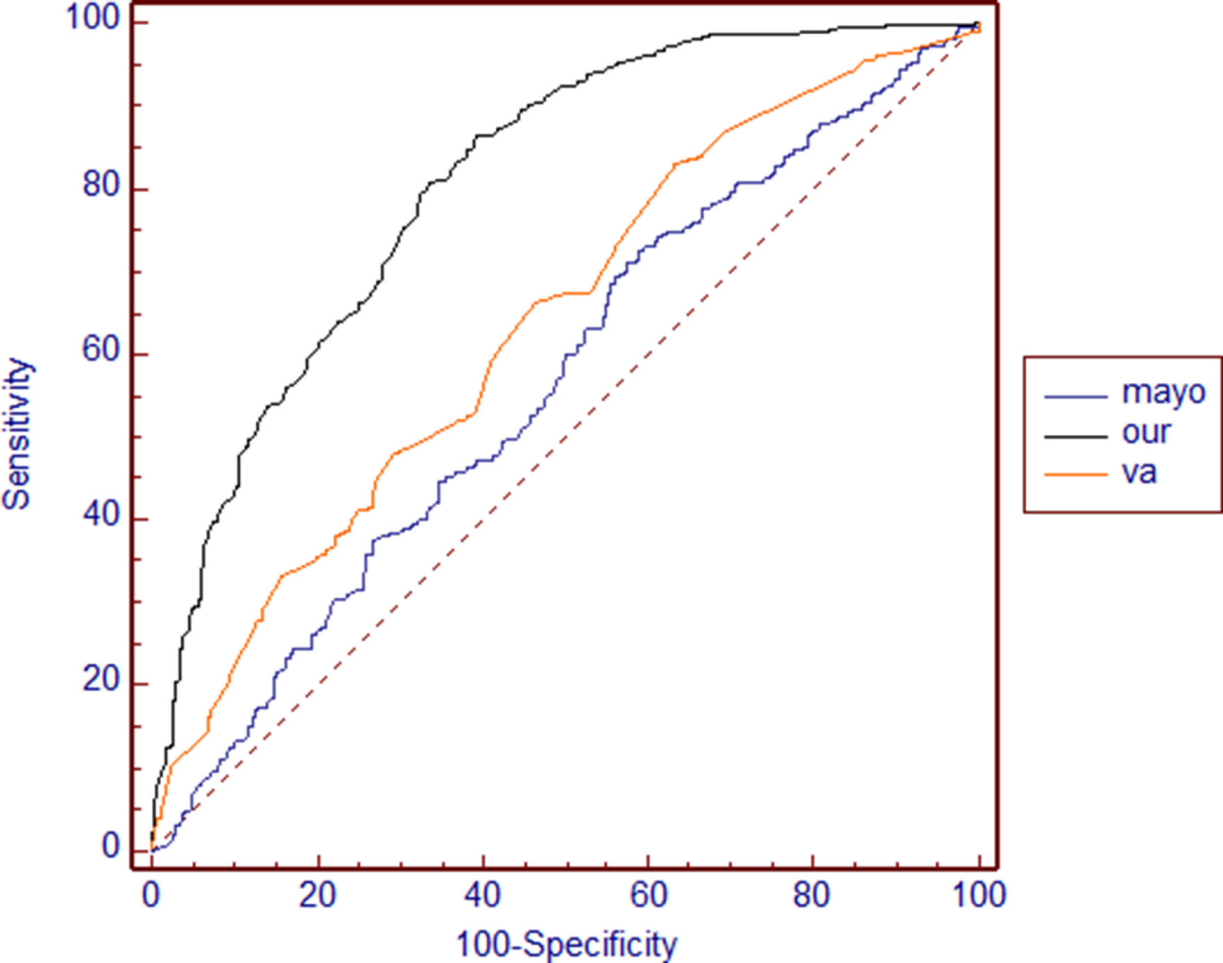
ROC curves of our model, the Mayo model and the VA model in the development data set

**Figure 3:**
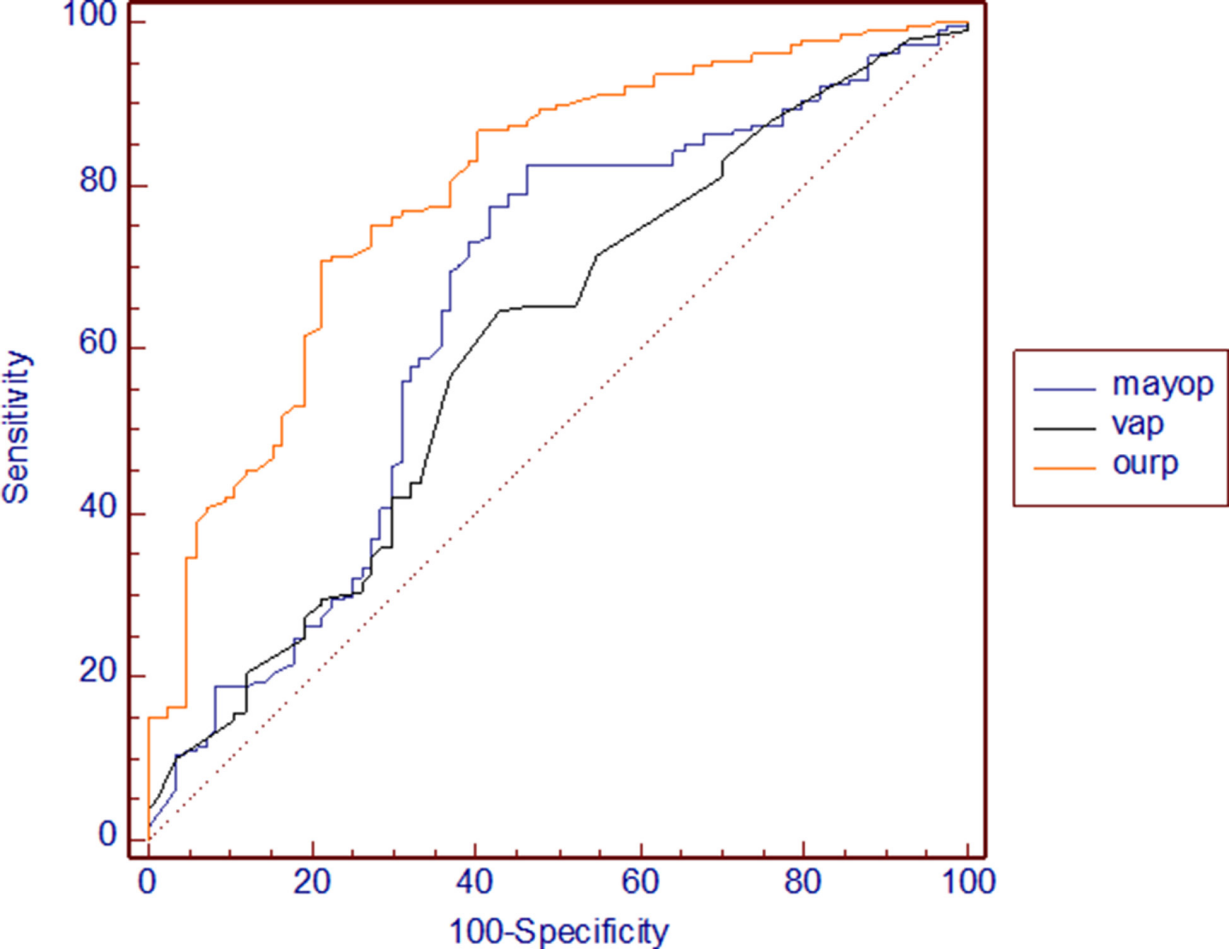
ROC curves of our model, the Mayo model and the VA model in the validation data set

The AUC values, sensitivity and specificity of our model in the development data set (0.807 ± 0.015, 95% CI 0.778–0.834, 85.71%, 60.36%) and validation data set (0.784 ± 0.027, 95% CI 0.731–0.831, 70.10%, 78.57%) were significantly different from those of the Mayo model (0.566 ± 0.022, 95% CI 0.53–0.6, 71.99%, 41.91%; 0.649 ± 0.037, 95% CI 0.59–0.706, 82.63%, 53.57%, *p* < 0.001) and VA model (0.636 ± 0.02, 95% CI 0.601–0.669, 66.11%, 53.01%; 0.599 ± 0.036, 95% CI 0.539–0.657, 63.40%, 57.14%, *p* < 0.001) (Figures 2, 3).

**Table:** 

Model	AUC	Standard Error	95%CI		Sensitivity Y	Specificity Y
			Lower	Upper		
Ours	0.807	0.015	0.778	0.834	85.71%	60.36%
Mayo	0.566	0.022	0.53	0.6	71.99%	41.91%
VA	0.636	0.02	0.601	0.669	66.11%	53.01%

**Table:** Comparison of ROC parameters in the development data set

	VA-Mayo		Ours-VA		Ours-Mayo	
Difference	0.07		0.171		0.242	
Standard error	0.027		0.021		0.023	
*p* value	0.008		< 0.001		< 0.001	

**Table:** 

Model	AUC	Standard Error	95%CI		Sensitivity Y	Specificity Y
			Lower	Upper		
Ours	0.784	0.027	0.731	0.831	70.10%	78.57%
Mayo	0.649	0.037	0.59	0.706	82.63%	53.57%
VA	0.599	0.036	0.539	0.657	63.40%	57.14%

**Table:** Comparison of ROC parameters in the validation data set

	VA-Mayo		Ours-VA		Ours-Mayo	
Difference	0.045		0.189		0.144	
Standard error	0.046		0.037		0.042	
*p* value	0.32		< 0.001		0.001	

### Complications

Among all the patients enrolled in this study (*n* = 1422), pneumothorax (16.53%), and hemorrhage of the lung (8.79%) or pleural cavity (4.08%) were the major complications of percutaneous lung biopsy. No cases of fatal air embolism or death occurred (Supplementary Table 2).

## DISCUSSION

Currently, the most common cause of cancer-related death is lung cancer [10]. Thus, early diagnosis and identification of pulmonary nodules has become increasingly important. The National Lung Screening Trial (NLST) found that although the rate of pulmonary nodule positivity was 25%, 96% of the nodules evaluated in that study were benign [11]. Therefore, the most important first step in the evaluation of pulmonary nodules is to assess the possibility of cancer and then choose the optimum diagnostic methods for evaluating the nodules. Currently, the recommendation for estimating the pretest probability of malignancy in all patients with SPNs, whether qualitatively on the basis of clinical evaluations or quantitatively using validated models, remains valid [7]. However, the sample sizes of the existing malignant risk prediction models for SPNs, such as the Mayo model, which included 419 cases [12]; the VA cooperative study, which included 375 cases [13]; the Herder model, which included 106 cases [14]; and the PKUPH model of China, which included only 107 cases, were small [15]. The Brock model included 1871 cases; however, most of them were cases involving multiple nodules, and the study comprised a lung-screening population in which less than 5% of nodules were found to be malignant [16]. Moreover, all models except the PKUPH model were developed using North American or European populations. Therefore, the most significant advantages of our study were its large SPN sample size and inclusion of a Chinese cohort.

In our model, age, malignancy history, pack-years of smoking and nodule size were the risk factors for malignant SPNs, and these factors were similar to those identified in previous models [12–16]. We found that upper lobe location was not a risk factor for malignant nodules in our model—a finding that contrasts with those regarding the risk factors for malignant nodules in the Mayo and Brock models [12, 16]—probably because tuberculosis is common in mainland China [17] and predominantly involves the upper lobe. Thus, we believe that the upper lobe is not a risk factor for malignant SPNs in countries with a high tuberculosis burden.

In the assessment of malignant lung nodules, nodule size was a significant, but not decisive [18–20]. However, the margins and contour of pulmonary nodules enable differentiation between benign and malignant nodules. Typical benign lesions have regular, smooth edges, while typical malignant nodules usually have lobulated, spiculated or irregular edges [21]. Swensen et al. found that a lobulated edge was an independent risk factor for malignant pulmonary nodules, with a positive predictive value of up to 88–94% [12]. In the Mayo and Brock models, spiculation was one of the risk factors associated with malignant pulmonary nodules [12, 14]; however, in the same models, as well as in the Herder model, lobulation was not a risk factor associated with malignant pulmonary nodules [12–14, 16]. We found that lobulation was associated with a greater risk for malignant pulmonary nodules than spiculation and that the greatest risk factor for malignant SPNs was an edge characterized by lobulation and spiculation (*p* < 0.001). Among our 118 SPN cases with lobulation and spiculation, the malignancy rate reached 94.07%, indicating that nodules with lobulation and spiculation greatly warrant increased clinical attention.

The greatest risk factor that was identified in our study but not in other studies, aside from the Brock model, was female gender. In contrast to most Western countries, whose lung cancer death rates are decreasing, China's lung cancer incidence rate is still increasing, as lung cancer remains the most commonly diagnosed cancer and is the leading cause of cancer-related death in this country [22–24]. Notably, the majority of affected non-smoking patients are women [25]. Freedman et al. reported that the incidence of lung cancer was higher in non-smoking women than in non-smoking men, although women were not more susceptible to the carcinogens present in tobacco smoke than men [26]. Evaluation of Pulmonary Nodules: Clinical Practice Consensus Guidelines for Asia recommend that practitioners should be aware of the risk of lung cancer caused by high levels of indoor and outdoor air pollution, as well as the high incidence of adenocarcinoma in female non-smokers [17]. NSCLC in non-smokers displays an increased clinical incidence in females and comprises a higher proportion of adenocarcinoma compared with NSCLC in ever smokers, both among surgical patients and among non-resectable advanced-stage patients [27]. Therefore, it is necessary to focus more attention on female patients with SPNs.

We established the largest sampling risk prediction model of SPNs in a Chinese cohort. SPNs in females, as well as SPNs featuring lobulated and spiculated edges or lobulation alone, should be evaluated more carefully because of the probability that they are malignant. The biggest advantage of our study was that all the lung nodules were solitary nodules, making it the largest study of SPNs assessing cancer risks in a Chinese cohort. A total of 98.25% of SPNs in our study were > 8 mm in size. Clinicians must assess such nodules immediately to determine if they are benign or have risk factors for malignancy to establish their clinical diagnoses. Our model is particularly applicable to SPNs > 8 mm in size. Almost all potential clinical and advanced imaging risk factors pertaining to malignant SPNs were incorporated into this model, along with a detailed stratification of several internal factors. The limitations of this study were that it was a single-center, retrospective study and that some of the patients had incomplete clinical data. A total of 161 patients (14.94%) with non-diagnostic results in the development data set failed to receive a final diagnosis. Additionally, the slice thickness of the images of the thorax that were obtained from Jan 1^st^ of 2011 to Dec 30^th^ of 2011 was 3 mm. Although these disadvantages may have led to bias that affected the results of our model, they are unavoidable and common problems in retrospective analyses. In the future, volume doubling times, blood-related indicators and genetic examination results will be incorporated into this assessment model to improve its accuracy [28–33].

## MATERIALS AND METHODS

### Participants and methods

We retrospectively reviewed the data from consecutive patients with SPNs who underwent CT-guided needle biopsy in our hospital from Jan 1st of 2011 to March 30th of 2016 (these data were obtained from the PACSKJLCT 08-SYSTEM, Chongqing, China). With the exception of patients with a history of primary lung cancer, no other patients were excluded from the study. A malignant pathologic diagnosis was based on an examination of tissue obtained via biopsy or surgery. A definitive benign diagnosis was established when a specific benign etiology was confirmed pathologically through biopsy or surgery, when an SPN was found to have been radiographically stable for at least 2 years or when an SPN had been clearly absorbed within a short time. SPNs that did not meet these criteria and patients who did not have follow-up data were classified as undiagnosed. All the patients were divided into two groups, a development data set (assessed from Jan 1st of 2011 to April 30^th^ of 2015) and a validation data set (assessed from May 1^st^ of 2015 to March 30^th^ of 2016). All patients underwent core needle biopsy using an Angiotech SuperCore™ Biopsy Instrument, 16 ga × 9 cm or ×15 cm (TECHNOLOGIES, USA), under the guidance of spiral CT (Siemens, Germany). CT was performed with single- (from Jan 1^st^ of 2011 to Oct 30th of 2015) or 16-detector (from Nov 1^st^ of 2015 to March 30th of 2016) CT scanners (tube voltage 120 kV, tube current 150 mA/ref, pitch 0.8). The datasets were derived from images of the thorax. The slices of the images obtained from Jan 1st of 2011 to Dec 30th of 2011 were 3 mm thick, and the remaining slices were 1 mm thick. The slices of the images obtained after biopsy were 3 mm thick. If the nodule was near the pulmonary hila or a blood vessel, we performed enhanced CT of the lesion before or during the biopsy. An average 1 to 3 needles were used during the puncturing process to successfully obtain the samples. All patients enrolled in this study had pathological diagnostic reports.

**Figure 4:**
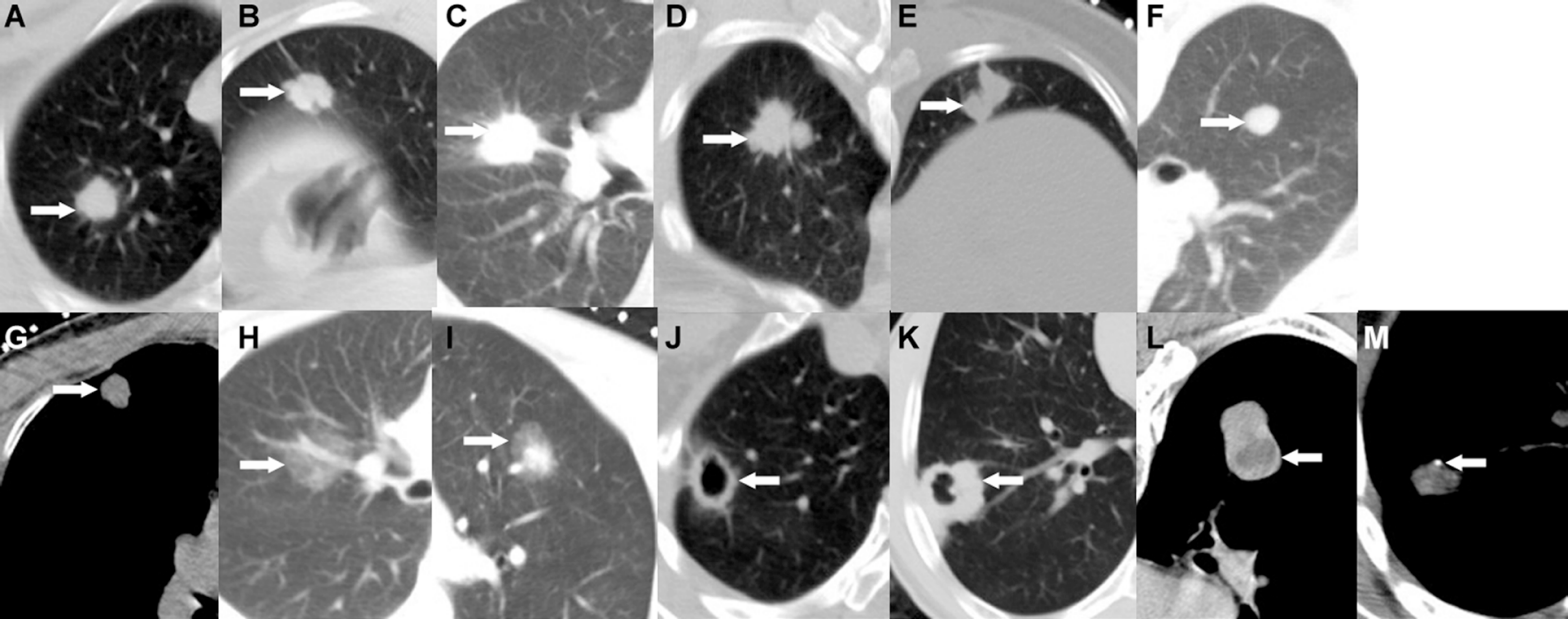
SPN edge and density characteristics (**A**) Spiculate protuberance, (**B**) Lobulation, (**C**) Spiculation, (**D**) Lobulation combined with spiculation, (**E**) Irregular edges, (**F**) Smooth edges, (**G**) Solid density, (**H**) Purely ground-glass, (**I**) Partly solid, (**J**) Thin cavitation, (**K**) Thickened cavitation, (**L**) Necrosis, (**M**) Calcification.

These reports included clinical data regarding sex, age, chief complaints, cigarette smoking status, previous medical histories and family histories (i.e., extra-thoracic disease history; chronic lung disease history, except cancer history; and cancer history, except lung cancer history), and histopathology. Information regarding the following chest radiological data was collected for all patients: nodule size (average of the maximum length and width) [8, 9], edge characteristics (i.e., whether the edges featured spiculated protuberances, lobulation alone, spiculation alone, or lobulation and spiculation; and whether the edges were irregular or smooth) [6], density characteristics (i.e., whether the nodules were solid, purely ground-glass, or partly solid; whether the nodules featured thin cavitations or thickened cavitations; and whether the nodules displayed necrosis and calcification) (Figure 4) and location (i.e., whether the nodules were located in the upper, middle or lower lobe). We obtained approval from our institution to use patient medical records for this study, and patient confidentiality was maintained.

### Statistical analysis

All statistical analyses, with the exception of those pertaining to the AUC graphs, which were performed using MedCalc, were conducted using SPSS ver. 20.0 (IBM). Significance was set at *p* < 0.05. Qualitative variables were expressed as absolute frequencies and percentages, and numerical variables were expressed as the mean ± SD. To assess the differences between the development data set and the validation data set, we analyzed numerical variables using independent samples *T*-tests, and we analyzed qualitative variables using a chi-square test.

We performed logistic regression analysis using potential predictors, including sex, age, chief complaint, cigarette smoking status, previous medical history, family history, nodule size, edge characteristics, density and location, to identify the risk factors for malignant SPNs. A prediction model was developed using the development data set and was validated with the validation data set. In the regression analysis, patients with non-diagnostic results were classified into the non-malignant group. We also compared each patient › s final diagnosis to the probability of malignancy predicted by the Mayo Clinic and VA models. We assessed model accuracy by calculating the AUCs of ROC. When comparing the performances of the two models, we included patients only if a score was available for each model.

## SUPPLEMENTARY MATERIALS FIGURES AND TABLES



## Abbreviations

SPN: Solitary pulmonary nodule
CT: Computed tomography
ACCP: American College of Chest Physicians
LDCT: Low-dose computed tomography
NLST: National Lung Cancer Screening Trial
ROC: Receiver operating characteristic
AUC: Area under the curve
PET-CT: Positron emission tomography and combined CT
