# Supplementary material for: Assessment of the cancer risk factors of solitary pulmonary nodules
Source: Oncotarget. 2017 Mar 21;8(17):29318–27. doi: 10.18632/oncotarget.16426 (PMC5438732; doi:10.18632/oncotarget.16426)
Supplement: Supplementary file 1 [file oncotarget-08-29318-s001.pdf]

Assessment of the cancer risk factors of solitary pulmonary nodules

Supplementary Materials

Supplementary Table 1: Other clinical characteristics of SPNs in development data set

Characteristics, *n* (%)	Total	Malignancy	Benign	Non-diagnosis
Participants	1078	721 (66.883%)	182(16.883%)	175(16.234%)
Chief complaint				
Censored data	165 (15.31%)	107 (14.84%)	23(12.64%)	35(20.00%)
Symptomatic patients	533	349 (48.40%)	95(52.20%)	89(50.86%)
Non-symptomatic patients	380	265 (36.75%)	64(35.16%)	51(29.14%)
Outpatients	303	214	37	52
Inpatients	775	507	145	123
Family history				
Censored data	654 (60.67%)	454 (62.97%)	92(50.55%)	108(61.71%)
No disease	295	174 (24.13%)	68(37.36%)	52(29.71%)
Extra-thoracic disease	4	4 (0.55%)	0	0
Lung disease excluding Malignancy	41	25 (3.47%)	10(5.49%)	6(3.43%)
Malignancy	84	63 (8.74%)	12(6.59%)	9(5.14%)

Supplementary Table 2: Complications of percutaneous lung biopsy

	**Total, n (%)**
Participant	1422
Pneumothorax (*n*, %)	235 (16.53%)
No	1183 (83.19%)
Pre operative	4 (0.28%)
A little	206 (14.49%)
Middle	17 (1.20%)
Severe or chest tube insertion	12 (0.84%)
Aerodermectasia (*n*, %)	14 (0.98%)
Hemorrhage (*n*, %)	
Lung	125 (8.79%)
A little	120 (8.44%)
Middle and above	5 (0.35%)
Pleural cavity	58 (4.08%)
A little	54 (3.80%)
Middle and above	4 (0.28%)
Serious complication* (*n*, %)	2 (0.14%)
Asphyxia	1 (0.07%)
Cerebral infarction	1 (0.07%)
Death	0

*The two patients were recovery after therapy.
